# Nonmetastatic pancreatic cancer: Improved survival with chemoradiotherapy > 40 Gy after systemic treatment

**DOI:** 10.1007/s00066-018-1281-7

**Published:** 2018-03-01

**Authors:** Sebastian Bachmayer, Gerd Fastner, Andrea Vaszi, Wolfgang Iglseder, Peter Kopp, Josef Holzinger, Adam Dinnewitzer, Gabriel Rinnerthaler, Simon Peter Gampenrieder, Klaus Emmanuel, Richard Greil, Felix Sedlmayer, Franz Zehentmayr

**Affiliations:** 1 Department of Radiotherapy and Radio-oncology, University Hospital Salzburg, Paracelsus Medical University, Salzburg, Austria; 2 radART Institute for Research and Development of Advanced Radiation Technologies, Paracelsus Medical University, Salzburg, Austria; 3 University Clinic of Surgery, University Hospital Salzburg, Paracelsus Medical University, Salzburg, Austria; 4 IIIrd Medical Department, University Hospital Salzburg, Paracelsus Medical University, Salzburg, Austria

**Keywords:** Pancreatic cancer, Adjuvant chemotherapy, Overall survival, Retrospective analysis, Surgery, Pankreaskarzinom, Adjuvante Chemotherapie, Gesamtüberleben, Retrospektive Analyse, Operation

## Abstract

**Purpose:**

The role of radiotherapy (RT) for nonmetastatic pancreatic cancer is still a matter of debate since randomized control trials have shown inconsistent results. The current retrospective single-institution study includes both resected and unresected patients with nonmetastasized pancreatic cancer. The aim is to analyze overall survival (OS) after irradiation combined with induction chemotherapy.

**Patients and methods:**

Of the 73 patients with nonmetastatic pancreatic cancer eligible for the present analysis, 42 (58%) patients had adjuvant chemoradiotherapy (CRT), while 31 (42%) received CRT as primary treatment. In all, 65 (89%) had chemotherapy at any time before, during, or after RT, and 39 (53%) received concomitant CRT. The median total dose was 50 Gy (range 12–77 Gy), while 61 (84%) patients received >40 Gy.

**Results:**

With a median follow-up of 22 months (range 1.2–179.8 months), 14 (19%) are still alive and 59 (81%) of the patients have died, whereby 51 (70%) were cancer-related deaths. Median OS and the 2‑year survival rate were 22.9 months (1.2–179.8 months) and 44%, respectively. In addition, 61 (84%) patients treated with >40 Gy had a survival advantage (median OS 23.7 vs. 17.3 months, *p* = 0.026), as had patients with 4 months minimum of systemic treatment (median OS 27.5 vs. 14.3 months, *p* = 0.0004).

**Conclusion:**

CRT with total doses >40 Gy after induction chemotherapy leads to improved OS in patients with nonmetastatic pancreatic cancer.

**Electronic supplementary material:**

The online version of this article (10.1007/s00066-018-1281-7) contains supplementary material, which is available to authorized users.

## Zusammenfassung

**Hintergrund:**

Die Bedeutung der Radiotherapie (RT) in der Behandlung des nichtmetastasierten Pankreaskarzinoms ist nach wie vor umstritten, zumal die Ergebnisse randomisierter Studien inkonklusiv sind. Die vorliegende retrospektive Studie inkludiert sowohl operierte als auch nichtoperierte Patienten mit nichtmetastasiertem Pankreaskarzinom. Primärer Endpunkt der Analyse ist das Gesamtüberleben (OS) nach Bestrahlung und vorangegangener Induktionschemotherapie.

**Patienten und Methoden:**

Analysiert wurden 73 Patienten mit nichtmetastasiertem Pankreaskarzinom. Von diesen hatten 42 (58 %) eine adjuvante Chemoradiotherapie (CRT). Eine CRT als primäre Behandlung erhielten 31 (42 %) nichtoperierte Patienten. Eine Chemotherapie vor, während oder nach RT hatten 65 (89 %), 39 (53 %) bekamen eine konkomittante CRT. Die mediane Gesamtdosis war 50 Gy (Spanne 12–77 Gy). Mehr als 40 Gy erhielten 61 Patienten (84 %).

**Ergebnisse:**

Bei einer medianen Nachsorgedauer von 22 Monaten (Spanne 1,2–179,8 Monate) waren 59 (81 %) Patienten verstorben, 14 (19 %) leben noch. Die Todesursache war bei 51 (70 %) Patienten ihre Tumorerkrankung. Das mediane Überleben betrug 22,9 Monate (Spanne 1,2–179,8 Monate), das 2‑Jahres-Überleben 44 %. Insgesamt 61 (84 %) Patienten, die mit >40 Gy behandelt wurden, hatten einen Überlebensvorteil (medianes OS 23,7 vs. 17,3 Monate; *p* = 0,026), ebenso Patienten nach mindestens 4 Monaten Systemtherapie (medianes OS 27,5 vs. 14,3 Monate; *p* = 0,0004).

**Schlussfolgerung:**

Eine CRT mit Gesamtdosen >40 Gy nach Induktionschemotherapie verbessert das OS bei Patienten mit nichtmetastasiertem Pankreaskarzinom.

## Introduction

In Europe, a total of 87,400 deaths due to pancreatic cancer were predicted for 2017, which amounts to approximately 6% of all cancer deaths [1]. These numbers indicate the aggressiveness of this disease and its dismal prognosis. Surgery is still the cornerstone of curative treatment. Unfortunately, only 20% of the patients are resectable at the time of diagnosis, 30% present with unresectable locally advanced disease, and 50% of the patients already have distant metastases [2].

While adjuvant chemotherapy (CT) is undisputed, the addition of radiotherapy is recommended only for subgroups of patients. According to the ASCO guidelines, it should be offered to patients with N1 and/or R1 status [3, 4], whereas in Germany adjuvant chemoradiotherapy (CRT) is restricted to clinical trials only [5]. The prospective studies that informed this conceptual framework were conducted between 1985 [6] and 2004 [7]. Based on an outdated radiation treatment schedule—40 Gy split course— the European Study Group for Pancreatic Cancer (ESPAC) came to the conclusion that CRT is not only inferior to CT but detrimental with respect to overall survival (OS). In the latest study, 50.4 Gy total dose in the CRT arm were administered, which resulted in OS rates similar to CT alone [8].

For the treatment of locally advanced pancreatic cancer (LAPC) guidelines recommend initial CT followed by CRT only for those patients without systemic progression [3, 4]. The prospective randomized control trials in the field published in the past decade [9–11] were conducted with radiation doses of 46 to 60 Gy. The study design of the latest of these three studies included a period of 4 months of CT before CRT with 54 Gy. The two treatment regimens were iso-effective with respect to OS but locoregional control and time to retreatment was significantly better in the CRT arm [11].

Taken together, the role of radiotherapy both for resectable and unresectable pancreatic cancer is still under investigation. Due to a probability of 30% for micrometastases at the time of diagnosis [12] patients with nonmetastasized pancreatic cancer may progress early at distant sites, which impairs clinical outcome. Simultaneously, locoregional recurrence may also lead to death [13].

The current study includes both resected and unresected patients with nonmetastasized pancreatic cancer. Its aim is to analyze OS following irradiation combined with induction chemotherapy in the therapeutic setting of a European tertiary referral center.

## Materials and methods

### Patients

Between 1982 and 2016, 150 patients with pancreatic and periampullary cancer were referred to our department for radiation therapy. In all, 77/150 (51%) patients were excluded from the present analysis mainly for distant metastasis at the time of diagnosis. For the current analysis we included 73 patients who were referred since 1998, when intraoperative radiotherapy with electrons (IOERT) as well as 3D CRT were fully implemented. The primary endpoint was overall survival (OS).

Patients’ characteristics are described in Table 1. Patients were staged according to the 6^th^ edition of the TNM system before 2010. When reviewing the charts in 2016 we used the 7^th^ edition as a reference knowing that the two systems were identical with respect to pancreatic cancer. The majority of patients were classified as T3 (55%) and T4 (38%), respectively. Out of T4 tumors, 14 (19%) patients presented with involvement of duodenum, spleen or adrenal glands. In total, 66/73 (90%) patients presented with Union internationale contre le cancer (UICC) stages IIB or III. In 47/73 (64%) cases, the primary tumor was located in the head of the pancreas or the uncinate process, in 4/73 (6%) the papilla of vater and in 19/73 (26%) in the body or tail. In addition, 3/73 (4%) were found in the neck of the pancreas. At the start of radiation treatment, 19/73 (26%) patients presented with local progression after induction CT. Disease progression was diagnosed by imaging (computed tomography, MRI or PET-CT scans) with histological confirmation at the discretion of the treating physician.

**Table 1:** Patient characteristics

Patient characteristics at baseline *N* = 73			
Sex (*n*)	Male	37	51%
	Female	36	49%
Age at diagnosis (years)	Median	66.9	–
	Range	45.6–83.7	–
Pathological confirmation	Yes	64	88%
	No	9	12%
Tumor location (*n*)	Papilla of vater	4	6%
	Head or uncinate process	47	64%
	Body or tail	19	26%
	Neck	3	4%
T (*n*)	T1	1	1%
	T2	4	5%
	T3	40	55%
	T4	28	38%
N (*n*)	N0	21	29%
	N1	52	71%
M (*n*)	M0	72	99%
	Mx	1	1%
Involvement of either duodenum, spleen or adrenal glands (*n*)	Yes	14	19%
	No	59	81%
Histologic type (*n*)	Papilla of vater	4	5%
	Adenocarcinoma	57	78%
	Neuroendocrine tumor	2	3%
	Unknown	10	14%
Grading (*n*)	G1	3	4%
	G2	38	52%
	G3	18	25%
	Gx	14	19%
UICC stage (*n*)	IA	1	1%
	IB	1	1%
	IIA	7	10%
	IIB	36	49%
	III	28	38%
Karnofsky performance score (*n*)	≥70	57	78%
	<70	16	22%
Local progression during CT before RT (*n*)	Yes	19	26%
	No	54	74%

*CT* chemotherapy, *RT* radiotherapy

### Treatment

In resectable patients (42/73, 58%), CRT was applied as adjuvant treatment, while being the primary approach for the 31/73 (42%) patients who presented with locally advanced disease deemed to be unresectable. Treatment characteristics are summarized in Table 2.

**Table 2:** Treatment characteristics

Treatment characteristics *N* = 73				
*Surgery*	Resection	Yes	42	58%
		No	31	42%
	Type of resection	Whipple	27	37%
		Other	15	21%
	Resection margin status	R0	20	27%
		R1	21	29%
		R2	1	1%
*Radiotherapy*	IOERT (*n*)	Yes	18	25%
		No	55	75%
	EBRT (*n*)	Yes	61	84%
		No	12	16%
		Median single dose (range)	1.8 Gy (1.6–2.0 Gy)	–
	EQD2 (EBRT + IORT)	Median total dose (range)	50 Gy (12–77 Gy)	–
		<40 Gy	12	16%
		≥40 Gy (*n*)	61	84%
*Chemotherapy*	Concomitant CRT	Yes	39	53%
		No	34	47%
	CT before RT	<4 months or none	29	40%
		>4 months	44	60%
	CT before, during or after RT	Yes	65	89%
		No	8	11%

*IOERT* intraoperative radiotherapy with electrons, *EBRT* external beam radiotherapy, *EQD2* biologically equivalent dose in 2 Gy fractions, *CT* chemotherapy, *RT* radiotherapy, *CRT* chemoradiotherapy

Surgery was performed in curative intention and resulted in 20/42 (48%) histologically complete (R0) and 21/42 (50%) incomplete (R1) resections. In 1/42 (2%) patient surgery was macroscopically incomplete (R2). Pathology reports were re-evaluated and—in accordance with Verbeke et al.—resection was regarded as incomplete if tumor cells were found within 1 mm of the resection margin [14].

Radiotherapy was performed as intraoperative radiotherapy with electrons (IOERT) and/or external beam treatment. IOERT was delivered by means of a standard LINAC (median 8 MeV, range 6–18 MeV). The energy was chosen according to dose depth (median 2.2 cm, range 0.9–4 cm) determined by ultrasound. The tube diameter was adjusted to tumor bed dimensions (median 3.7 cm, range 2.0–5.5 cm). The median 90% isodose was 9 Gy (range 7–18 Gy). A total of 18/73 (24%) patients received single fraction IOERT during surgery, and out of these, 3 patients in the course of explorative laparotomies. The median IOERT dose (Dmax) was 10 Gy (range 8–20 Gy), resulting in a median total equivalent dose (EQD2) of 17 Gy (range 12–49 Gy) in the tumor bed.

EBRT was usually performed with 15 MV photons (range 15–25 MV) in conventional 3D-box technique (three or four portals). The median single dose was 1.8 Gy (range 1.6–2 Gy). The dose–volume constraints were set at the usual levels with maximum doses of small intestine as well as myelon at 45 Gy, the stomach at 50 Gy, V50 for the kidney <30% [15]. A total of 61/73 (84%) patients received EBRT, one of these was treated with IMRT. In 43/73 (59%) patients, the planning target volume included the regional lymphatic drain. The median total EQD2 (EBRT + IOERT) for all patients was 50 Gy (range 12–77 Gy); 61/73 (84%) patients received more than 40 Gy EQD2 (median 50 Gy, range 44–77 Gy).

The chemotherapeutic regimens were based on 5‑FU (or capecitabine) or gemcitabine. In all, 26/42 resected patients received CT before RT in two possible therapy sequences: CT + surgery + (C)RT (*n* = 2) or surgery + CT + (C)RT (*n* = 24). Thus, induction chemotherapy in the strict sense was applied in 2 cases with the intention to achieve operability. A total of 18 patients had gemcitabine mono (1000 mg/m^2^ on day 1, 3 out of 4 weeks), 3 GEMOX (gemcitabine 1000 mg/m^2^, oxaliplatin 100 mg/m^2^, every 2 weeks), 3 FOLFIRINOX (oxaliplatin 85 mg/m^2^, irinotecan 180 mg/m^2^, leucovorin 400 mg/m^2^, 5‑FU bolus 400 mg/m^2^, 5‑FU continuous 2400 mg/m^2^ over 46 h, every 2 weeks), one patient had 5‑FU/leucovorin (425 mg/m^2^, 20 mg/m^2^, weekly) and another one EVANS (doxorubicin 50 mg/m^2^ on day 1, cyclophosphamide 1000 mg/m^2^ on day 1, cisplatin 25 mg/m^2^ on days 1–5, etoposide 50 mg/m^2^ on days 1–5, every 3 weeks). The median number of cycles was 6 (range 1–17). In the group of unresected patients 27/31 had CT before RT: 6 gemcitabine mono, 10 GEMOX, 10 FOLFIRINOX and one EVANS. The median number of cycles was 4 (range 3–11).

CRT was administered to 39/73 (54%) patients, while 65/73 (89%) patients had chemotherapy at any time (prior, following or concomitant to irradiation).

### Equivalent total dose in 2 Gy fractions (EQD2)

The generally accepted α/β values for gastrointestinal tissues range from 6–13 Gy [16, 17]. A recent publication by Unkel et al. analyzed clonogenic assays in nine pancreatic cancer cell lines. The median α/β value extrapolated from these experiments was 9.4 Gy (range 1.85–191.58 Gy; [18]). Taking into account the considerable heterogeneity of these numbers EQD2 in the current study was calculated with an α/β value of 10.

### Statistics

OS was defined as the time from diagnosis until death or last follow-up and estimated by the Kaplan–Meier method. The patient cohort was divided into groups according to EQD2: dose splits were set at 40 Gy, 45 Gy, 50 Gy, and 55 Gy. These subgroups were compared by log-rank testing. In order to detect patient-related factors that are prognostically relevant with respect to OS, multivariate analyses (MVA) were performed by Cox regression (forward likelihood ratio). MVA models were calculated for resected and unresected patients separately. They included the following variables: sex, age at diagnosis, tumor location, T‑stage, N‑stage, involvement of adjacent structures (i.e., duodenum, spleen, adrenal glands), histology, grading, UICC stage, Karnofsky performance score, local progression before RT. *P*-values of 0.05 with a 95% confidence interval were regarded as statistically significant. Data were analyzed with SPSS™ for Windows v 21.0.0.1.

## Results

**Fig. 1:**
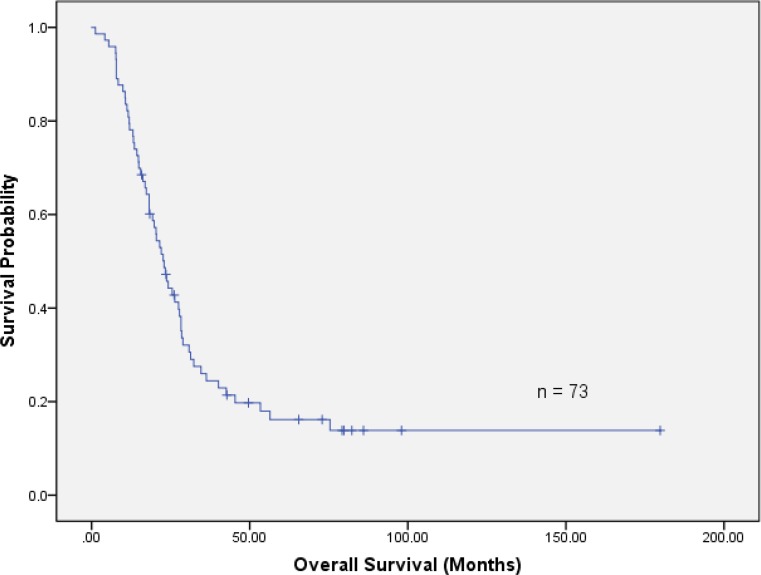
Overall survival in 73 patients with nonmetastatic pancreatic cancer

With a median follow-up of 22 months (range 1.2–179.8 months), 59/73 (81%) of the patients died and 14/73 (19%) are still alive. There were 51/73 (70%) cancer-related deaths, 1/73 (1%) patient died for septic multiple organ failure and 2/73 (2%) died for cardiac reasons. In 5/73 (7%) patients, the medical charts were incomplete and no information could be obtained so that the cause of death remained unknown. The median OS for the whole patient cohort was 22.9 months, with 2‑ and 5‑year survival rates of 44 and 16%, respectively (Fig. 1). In the subgroup of resected patients, the corresponding numbers were 25.5 months, 52 and 20%, while in unresected patients median survival was 20.4 months, 2‑year and 5‑year survival rates were 34 and 13%, respectively (log-rank *p* = 0.154).

**Fig. 2:**
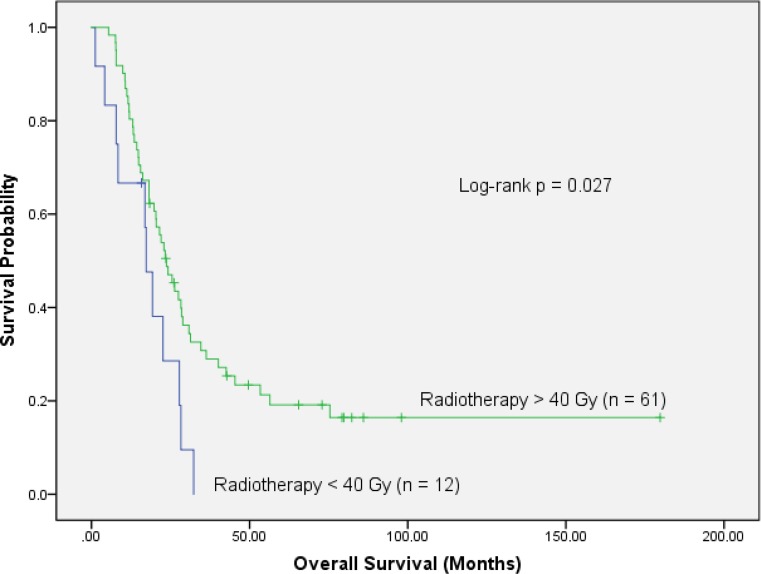
Overall survival is significantly improved in 61 patients with radiotherapy doses >40 Gy compared to the 12 patients who received <40 Gy (log-rank *p* = 0.027)

Based on EQD2, patients were grouped into two dose bins with a threshold dose below (group 1) or above 40 Gy (group 2), respectively. Fig. 2 shows the comparison of 61/73 (84%) patients in group 1 who were treated with at least 40 Gy EQD2 (median 50 Gy, range 44–77 Gy) compared to 12/73 (16%) individuals (group 2) who received less than 40 Gy (median 17 Gy, range 12–37 Gy) for one of the following reasons: postsurgery complications (5), lack of compliance (2), M1 during chemotherapy (3), multiorgan failure due to infection (1). Additionally, one patient received previous irradiation for seminoma 26 years before he was diagnosed with pancreatic cancer. In group 1, the median OS was 23.7 months, and the 2‑ and 5‑years survival rates amounted to 47 and 19%, while in group 2 the respective numbers were 17.3 months, 19 and 0% (*p* = 0.026). Of note, this difference remained significant within the cohort of the 42 patients who had received surgery: when treated with doses >40 Gy, the median survival time, as well as the 2‑ and 5‑year survival rates amounted to 27.5 months, 56 and 20%, respectively, compared to 19.3 months, 23 and 0% in those patients with the lower dose (*p* = 0.028). For the 31 unresected patients no significant difference could be detected (*p* = 0.073): patients with >40 Gy had a median, 2‑ and 5‑years survival of 20.4 months, 37 and 14%, compared to 4.2 months, 0% for those with lower doses.

**Fig. 3:**
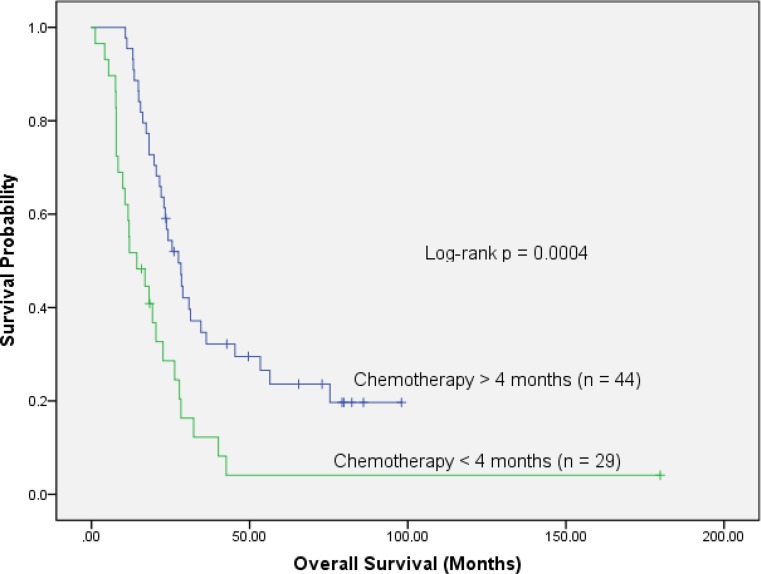
Comparison of patients by duration of induction chemotherapy: overall survival is significantly better in 44 patients with >4 months than in 29 patients with <4 months (log-rank *p* = 0.0004)

We also compared survival between patients with 4 months of chemotherapy to those without. Patients with at least 4 months of systemic treatment lived significantly longer than those who had less (>4 months: median OS 27.5 months, 2 year 54%, 5 year 24%; <4 months: median OS 14.3 months, 2 year 25%, 5 year 4%; *p* = 0.0004, Fig. 3). This significant difference persisted in resected patients (*p* = 0.003, supplementary Fig. 1) as well as in unresected patients (*p* = 0.023, supplementary Fig. 2).

**Fig. 4:**
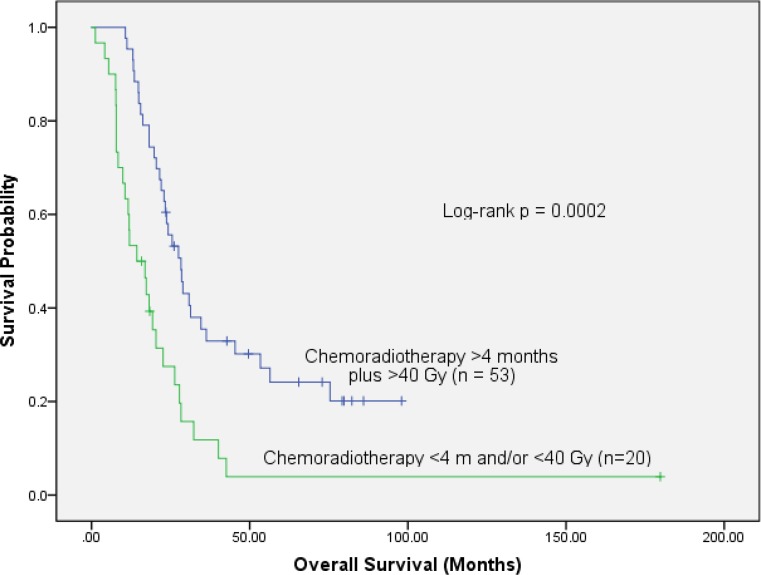
Comparison by combined treatment modalities: overall survival is significantly better in 53 patients who received >4 months of chemotherapy followed by >40 Gy than in the other 20 patients who had <4 months of chemotherapy and/or <40 Gy (log-rank *p* = 0.0002)

A comparison of combined treatment modalities showed that patients who received >4 months of systemic treatment followed by >40 Gy radiation therapy had an overall survival advantage (median OS 28.3 months versus 14.3 months, *p* = 0.0002, Fig. 4). Again, the difference persisted both in resected (*p* = 0.003) and unresected patients (*p* = 0.020).

Prognostically relevant parameters for overall survival were analyzed separately for resected and unresected patients (Table 3). In resected patients involvement of either duodenum, spleen or adrenal glands was a significant factor in univariate analysis (*p* = 0.046) but no longer in the MVA model. In unresected patients, local progression was found to be a significant prognosticator in both models (UVA: *p* = 0.014; MVA: HR = 4.132, 95% CI 1.217–14.030, *p* = 0.023).

**Table 3:** Prognostic factors for overall survival

Prognostic factors				
	Resected patients		Unresected patients	
	UVA	MVA	UVA	MVA
Sex	0.091	n.s.	0.376	n.s.
Age at diagnosis	0.974	n.s.	0.660	n.s.
Tumor location	0.287	n.s.	0.846	n.s.
T	0.269	n.s.	0.992	n.s.
N	0.226	n.s.	0.162	n.s.
Involvement of either duodenum, spleen or adrenal glands	0.046	n.s.	0.778	n.s.
Histologic type	0.127	n.s.	0.634	n.s.
Grading	0.051	n.s.	0.300	n.s.
UICC stage	0.088	n.s.	0.634	n.s.
Karnofsky performance score	0.629	n.s.	0.737	n.s.
Local progression during induction chemotherapy	0.726	n.s.	0.014	0.023

*UVA* univariate analysis, *MVA* multivariate analysis, *n.s.* not significant

Toxicity was mild and well manageable: 67/73 (92%) patients had no major adverse effects reported, while 6/73 (8%) patients suffered from radiation induced inflammatory reactions (gastritis, duodenitis, colitis, inflammation of anastomosis).

## Discussion

In this retrospective analysis we could demonstrate that following a minimum of 4 months of systemic treatment, the administration of a total dose higher than 40 Gy improves OS in patients with nonmetastatic pancreatic cancer.

A total of 40 Gy was selected as cutoff since practice changing studies conducted by ESPAC [7], the European Organisation for Research and Treatment of Cancer (EORTC) [19] and the Gastrointestinal Tumor Study Group (GITSG) [6] used this regimen (40 Gy in 2 Gy fractions, split course). This amounts to an EQD2 of 32 Gy assuming a daily loss of 0.6 Gy during the 2‑week break. The median dose of the study published by the Groupe Coopérateur Multidisciplinaire en Oncologie (GERCOR) was 50.4 Gy conventional RT [8]. With the radiation regimens implemented in these trials, median OS ranges between 159 [7] and 243 [8] months were achieved. In retrospective series and population-based cohort studies, the highest reported median OS was 39.9 months with CRT at median total doses of 50.4 Gy [20–30]. A National Cancer Database (NCDB) analysis revealing a median OS of 21 months concluded that the optimal dose range for adjuvant RT is possibly between 50 and 55 Gy, while doses <40 Gy lead to worse survival [24]. In the current study 31/42 (74%) of the resected patients received >40 Gy. With this treatment schedule, a median OS of 25.5 months was achieved, which is on the upper edge of the published results for CRT arms of prospective studies [6–8, 19]. Except for the GERCOR trial, these studies applied radiation in split course to potentially large volumes without a centralized quality audit. Apart from the RT concepts, these studies were frequently criticized for their design and statistical analyses so that their results remain disputed.

Adjuvant CT trials report mOS between 22.1 and 28 months [31–34]. Except for one outlier of 48 months [35] this is well in line with our results. A re-analysis of the ESPAC-3 data concluded that the administration of six cycles of adjuvant CT is more important in terms of OS than an early start after surgery in order to allow for complete recovery [36]. On average the resected patients in our cohort received six cycles of adjuvant CT and therefore fulfill this prerequisite for the best possible outcome.

A recent comprehensive review including four meta-analyses [37–40] stated that despite of the inconclusiveness of prospective and retrospective studies, adjuvant CRT should be offered to patients with N1 and/or R1 situation after 4–6 months of chemotherapy [4, 30]. Our data corroborate the idea that 4 months of CT prior to RT improves OS (Fig. 3), whereas neither resection nor lymph node status were significant prognosticators in our cohort. A point of criticism in the above mentioned meta-analyses [37–40] is the disregard for improved local control by CRT. Of note, especially in primarily resectable patients who received adjuvant CT after surgery, the rate of isolated local relapses lies between 18 and 28% [33–35]. This is also true for the CT arm of the EORTC-40013-22012/FFCD 9203/GERCOR study with 24% isolated local relapses reported, compared to 11% in the CRT arm [8]. This underlines the importance of RT as an effective adjunct local treatment, which reduces the rate of local relapses and their consequences (pain, lower quality of life, higher rate of retreatment) by a factor of 2. When considering also patients with synchronous distant metastases, the percentage of patients who experience a local relapse is even higher (34 to 82%; [31, 33, 34]). These figures clearly challenge the widely spread notion that CRT should not play a role in resectable pancreatic cancer. In contrast to this assumption, we strongly advocate RT as a powerful tool for the prevention of a local regrowth.

In LAPC patients the mOS in the present analysis was roughly 20 months, which seems to be superior to the mOS rates reported in the majority of clinical trials (8.3–17.4 months; [9–12, 41–43]). Being aware of the fact that a comparison with prospectively collected data is indirect (supplementary Table 2) a critical discussion of our findings has to consider patient selection as well as therapy regimens and toxicity. Reportedly, up to 30% of the patients develop metastases during induction CT and are therefore considered as unsuitable for subsequent combined CRT [12]. In trials without preceding CT, these patients are however included in the CRT group, which may compromise mOS [9, 10]. In our cohort of unresected patients, 27/31 (90%) received systemic treatment before CRT, thus selecting those patients who would potentially benefit most from RT.

CRT was administered in 19 cases mostly as single agent therapy (either gemcitabine or 5‑FU). Therefore, toxicity might be higher in prospective studies that apply CT doublets concomitant to irradiation. For example, in the FFCD/SFRO study only 40% of the patients received CRT as planned. The mOS was 13 months with gemcitabine mono versus 8.6 months with 60 Gy plus 5‑FU/cisplatinum [9]. Hence, the differences between the current study and prospective trials may—on the one hand—be due to the effectiveness of systemic treatment before CRT, which selects those patients who are most suitable for locoregional treatment. On the other hand, single agent CRT entails a lower rate of toxicity related treatment interruptions. Hence, in terms of mOS, a less aggressive strategy might be of advantage in this generally frail patient population.

The LAP07 study is the latest prospective randomized control trial on LAPC [11]. After the first randomization 223 patients received gemcitabine weekly and 219 patients the same CT combined with erlotinib. The second randomization step allocated those patients without tumor progression either continue the same systemic treatment (136 patients) or to receive 54 Gy CRT with capecitabine (133 patients). Median OS, which was the primary endpoint, did not differ significantly between the CRT and the CT arm (15.2 and 16.5 months; [11, 44]). In the current study, 30/31 (97%) patients with unresectable LAPC received >40 Gy, which results in a median OS of 20.4 months and 2‑year survival rates of 34%. At first sight, our findings seem to be in contrast to the results of the LAP07 study, where no survival difference was noted between patients receiving CRT with 54 Gy versus CT only. However, LAP07—like similar studies listed in supplementary Table 2—tested CRT versus CT alone, whereas the current retrospective analysis compares different dose levels (> vs. <40 Gy) within a cohort treated with RT. Thus, since the treatment characteristics of patient groups are different in these analyses, a direct comparison is—to our mind—hardly possible.

The current study is limited by its retrospective nature and the small sample size with 73 patients included between 1998 and 2016. The number of CRT patients in prospective studies is generally small compared to CT trials both in resectable and unresectable situations. In trials with postoperative CRT it is between 21 and 145 patients [6–8, 19] (supplementary Table 1). In 2/4 (50%) of these studies [6, 8] the number of CRT patients included is smaller than in the current cohort. Considering only resected patients in our cohort (*n* = 42), there is still one study that is smaller [6] and another one with only three patients more than in our collective [8]. In prospective LAPC studies the total number of patients is 23 to 109, 6/8 (75%) studies include less than the current study. The number of unresected patients in our cohort is 31, in 2/8 (25%) of the mentioned prospected LAPC trials it is lower ([9–12, 42, 43, 45, 46]; supplementary Table 2). Admittedly, a downside of the current analysis is that the group sizes are not well balanced, which reflects daily clinical practice.

Due to the inherent selection bias in retrospective studies disproportionate group sizes may lead to statistical inaccuracies so the results have to be interpreted with caution. The statistics should be understood as largely descriptive of a heterogeneous patient population and cannot be taken as a basis to draw firm conclusions. In addition, patients were treated with a variety of CRT schedules, which reflects clinical practice. Also, there are no clear cutoff criteria for more or less than 4 months of systemic treatment before CRT. This decision was left at the discretion of the treating medical oncologist and discussed in the tumor board. The retrospective application of a 4-month cutoff in our cohort was based on published literature rather than on a strict study design per se [11].

Despite these shortcomings our data confirm that CRT with threshold doses >40 Gy after at least 4 months of CT is beneficial both for resected and unresected patients with nonmetastasized pancreatic cancer.

## Caption Electronic Supplementary Material

Supplementary figure 1. Overall survival in resected patients compared by duration of systemic treatment before RT

Supplementary figure 2. Overall survival in unresected patients compared by duration of systemic treatment before RT

Supplementary table 1. Overview of prospective, retrospective and population-based studies

Supplementary table 2. Prospective studies for LAPC

## Abbreviations

ASCO: American Society of Clinical Oncology
CT: Chemotherapy
CRT: Chemoradiotherapy
EBRT: External beam radiotherapy
EORTC: European Organisation for Research and Treatment of Cancer
ESPAC: European Study Group for Pancreatic Cancer
EQD2: Biologically equivalent dose in 2 Gy fractions
GERCOR: Groupe Coopérateur Multidisciplinaire en Oncologie
GITSG: Gastrointestinal Tumor Study Group
HR: Hazard ratio
IMRT: Intensity-modulated radiotherapy
IOERT: Intraoperative radiotherapy with electrons
LAPC: Locally advanced pancreatic cancer
mOS: Median overall survival
mRT‑dose: Median radiation dose
MVA: Multivariate analysis
NCDB: National Cancer Database
NR: Not reported
NS: Not significant
OS: Overall survival
RT: Radiotherapy
UICC: Union Internationale Contre le Cancer
