# Supplementary figures and images for: Nonmetastatic pancreatic cancer: Improved survival with chemoradiotherapy > 40 Gy after systemic treatment
Source: Strahlenther Onkol. 2018 Mar 1;194(7):627–37. doi: 10.1007/s00066-018-1281-7 (PMC6008353; doi:10.1007/s00066-018-1281-7)

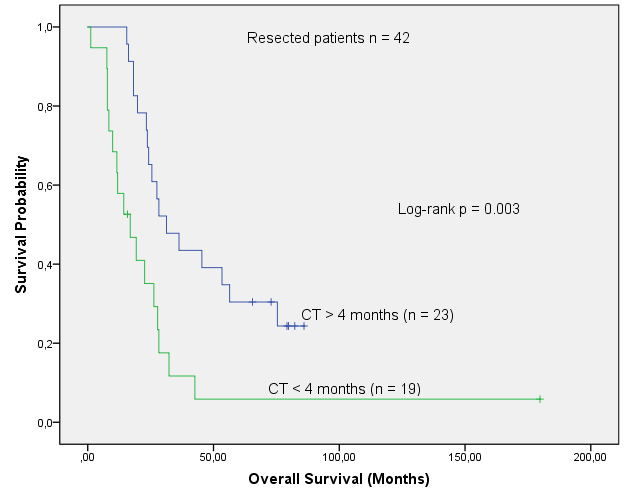

Supplement: Supplementary file 1 — Supplementary figure 1. Overall survival in resected patients compared by duration of systemic treatment before RT [file 66_2018_1281_MOESM1_ESM.docx]

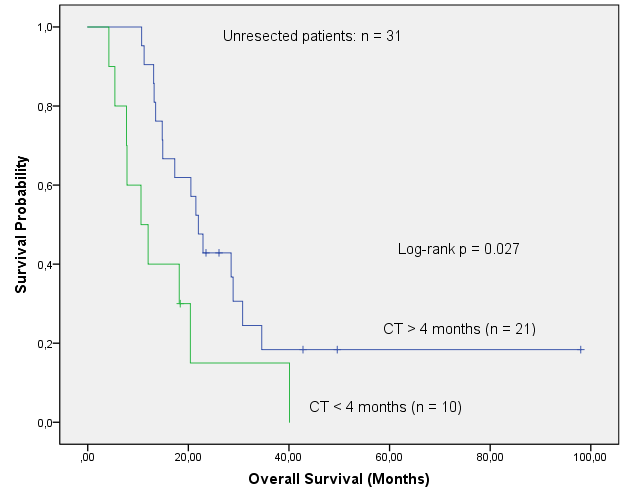

Supplement: Supplementary file 2 — Supplementary figure 2. Overall survival in unresected patients compared by duration of systemic treatment before RT [file 66_2018_1281_MOESM2_ESM.docx]
