# Supplementary material for: Nonmetastatic pancreatic cancer: Improved survival with chemoradiotherapy > 40 Gy after systemic treatment
Source: Strahlenther Onkol. 2018 Mar 1;194(7):627–37. doi: 10.1007/s00066-018-1281-7 (PMC6008353; doi:10.1007/s00066-018-1281-7)
Supplement: Supplementary file 3 — Supplementary table 1. Overview of prospective, retrospective and population-based studies [file 66_2018_1281_MOESM3_ESM.docx]

Supplementary table 1. Overview of prospective, retrospective and population-based studies in resected and unresected patients with non-metastatic pancreatic cancer: mOS = median overall survival, RT = radiotherapy, mRT-dose = median radiation dose, NR = not reported, NS = not significant, *number not directly mentioned in the cited paper but read off the Kaplan-Meier-plots in the results sections, °abstract only, §split course, ^the non-RT arm was best supportive care.

| **Study** | | | **N** | **mOS with RT (months)** | **mOS without RT (months)** | **2-year OS with RT** | **2-year OS without RT** | **p-value** | **mRT-dose (Gy)** |
| --- | --- | --- | --- | --- | --- | --- | --- | --- | --- |
|  |  |  |  |  |  |  |  |  |  |
| **Resected patients** | **Prospective** | GITSG 1985 | 43 | 21.0 | 10.9 | 43% | 18% | 0.03 | 40§ |
|  |  | EORTC 1999 | 207 | 24.5 | 19 | 51% | 41% | 0.208 | 40§ |
|  |  | ESPAC 2004 | 289 | 15.9 | 17.9 | 29% | 41% | 0.05 | 40§ |
|  |  | GERCOR 2010 | 90 | 24.3 | 24.4 | 51% | 50% | NR | 50.4 |
|  | **Retrospective** | Sohn 2000 | 616 | 19* | 20* | 38%* | 28%* | <0.0001 | NR |
|  |  | Hermans 2008 | 616 | 21.2 | 14.4 | 44% | 32% | <0.001 | 50 |
|  |  | Corsini 2008 | 466 | 25.2 | 19.2 | 50% | 39% | 0,001 | 50.4 |
|  |  | Morganti 2014 | 955 | 39.9 | 27.8 | 75%* | 50%* | <0.001 | 50.4 |
|  |  | **Current study** | **42** | **25.5** | **NR** | **52%** | **NR** | **NR** | **50** |
|  | **Population-based** | Hall 2013 | 1385 | 21.0 | NR | 40%* | NR | NR | 45 |
|  |  | Kooby 2013 | 11526 | 21* | 19* | 38% | 34% | <0.001 | NR |
|  |  | Sugawara 2014 | 2532 | 20.0 | 16.0 | 34%* | 30%* | <0.0001 | NR |
|  |  | Lim 2015 | 445 | 20.0 | 15.0 | 43% | 31% | 0.012 | NR |
|  |  | Mellon 2015 | 2966 | 21.0 | 20.0 | 35%* | 27%* | 0.02 | NR |
|  |  | Rutter 2015 | 6165 | 22.3 | 20.0 | 45% | 41% | <0.01 | 50.4 |
|  |  | Xia 2016 | 5304 | 19.0 | 14.0 | NR | NR | <0.001 | NR |
| **Unresected patients** | **Prospective** | Hazel 1981 | 30 | 7.3 | 7.8 | NR | NR | NS | 46 |
|  |  | Klaassen 1985 | 91 | 8.3 | 8.2 | NR | NR | NS | 40 |
|  |  | GITSG 1988 | 43 | 10.5 | 8 | NR | NR | 0.02 | 54 |
|  |  | Shinchi 2002^ | 31 | 13.2 | 6.4 | 0% | 0% | < 0.001 | 50.8 |
|  |  | Chauffert 2008 | 119 | 8.6 | 13 | 15%* | 21%* | 0.03 | 60 |
|  |  | Loehrer 2011 | 71 | 11.1 | 9.2 | 12% | 5% | 0.017 | 46 |
|  |  | Hammel 2016 | 269 | 15.2 | 16.5 | 25%* | 22%* | NS | 54 |
|  | **Retrospective** | Krishnan 2007 | 323 | 9 | NR | 9% | NR | NR | 30 |
|  |  | Huguet 2007 | 181 | 15 | 11.7 | 25%* | 10%* | 0.0009 | 55 |
|  |  | Ioka 2010° | 80 | 13 | 12.4 | NR | NR | 0.02 | 50 |
|  |  | **Current study** | **31** | **20.4** | **NR** | **34%** | **NR** | **NR** | **50** |
