# Supplementary material for: Nonmetastatic pancreatic cancer: Improved survival with chemoradiotherapy > 40 Gy after systemic treatment
Source: Strahlenther Onkol. 2018 Mar 1;194(7):627–37. doi: 10.1007/s00066-018-1281-7 (PMC6008353; doi:10.1007/s00066-018-1281-7)
Supplement: Supplementary file 4 — Supplementary table 2. Prospective studies for LAPC [file 66_2018_1281_MOESM4_ESM.docx]

Supplementary table 2. Prospective studies for LAPC: comparison of patient and tumor characteristics, study design and median overall survival in prospective studies and the current cohort.

| **Current study N = 31** | | | | **Wilson 2016** | **Hammel 2016** | **Mukherjee 2013** | **Loehrer 2011** | **Chauffert 2008** | **Moureau-Zambotto 2008** | **Huguet 2007** | **Louvet 2002** |
| --- | --- | --- | --- | --- | --- | --- | --- | --- | --- | --- | --- |
| **Sex** | male | 12 | 39% | 40% | 40% | 50% | 50% | 55% | 50% | 60% | 60% |
|  | female | 19 | 61% | 60% | 60% | 50% | 50% | 45% | 50% | 40% | 40% |
| **Age** | median | 69,5 |  | 65 | 63 | 64 | 66 | 60 | 60 | 61 | 60 |
| **Location** | head | 19 | 61% | n.a. | 70% | 85% | 60% | 70% | 80% | 65% | n.a. |
|  | body/tail | 14 | 45% | n.a. | 30% | 15% | 40% | 30% | 20% | 35% | n.a. |
| **KPS** | ≥70 | 22 | 71% | 100% | 95% | 95% | 100% | 85% | 95% | 85% | n.a. |
|  | <70 | 10 | 32% | n.a. | 5% | 5% | 0% | 15% | 15% | 15% | n.a. |
| **T** | <T3 | 1 | 3% | n.a. | n.a. | n.a. | n.a. | n.a. | n.a. | n.a. | n.a. |
|  | T3/T4 | 30 | 97% | n.a. | n.a. | n.a. | n.a. | n.a. | n.a. | n.a. | n.a. |
| **N** | N0 | 15 | 48% | n.a. | 60% | n.a. | n.a. | 60% | n.a. | n.a. | n.a. |
|  | N1 | 16 | 52% | n.a. | 40% | n.a. | n.a. | 40% | n.a. | n.a. | n.a. |
| **Grading** | G1 | 1 | 3% | n.a. | 24% | n.a. | 14% | n.a. | n.a. | n.a. | n.a. |
|  | G2 | 11 | 35% | n.a. | 17% | n.a. | 20% | n.a. | n.a. | n.a. | n.a. |
|  | G3 | 6 | 19% | n.a. | 7% | n.a. | 17% | n.a. | n.a. | n.a. | n.a. |
|  | Gx | 13 | 42% | n.a. | 50% | n.a. | 50% | n.a. | n.a. | n.a. | n.a. |
| **Upfront CRT** | | no | | yes | no | no | yes | yes | no | no | no |
| **OS (months)** | | median | 20,4 | 17,4 | 16,5 | 15,2 | 11,1 | 13 | 12,6 | 13,1 | 11,5 |
|  |  | range | 13,0 - 28,0 | 12,8 - 18,8 | 14,5 - 18,5 | 13,9 - 19,2 | 7,6 - 15,5 | 8,7 - 18,1 | n.a. | n.a. | n.a. |
